# Electrophysiological Mechanisms Underlying Time-Dependent Assessments in Moral Decision-Making

**DOI:** 10.3389/fnins.2019.01021

**Published:** 2019-09-20

**Authors:** Jin Ho Yun, Jing Zhang, Eun-Ju Lee

**Affiliations:** ^1^Business School, Sungkyunkwan University, Seoul, South Korea; ^2^Convergence Institute for Intelligence and Informatics, Suwon, South Korea

**Keywords:** neuroeconomics, moral decision-making, gain-loss asymmetry, event-related potential, movement-related potential

## Abstract

Human decision-making that involves moral dilemmas is a complex process, as individuals try to adhere to their moral values while their actual decisions can be influenced by several situational constraints. When facing a moral conflict that can bring a gain or loss for a decision-maker but a corresponding loss or gain for others, the decision-maker’s choice of resolution strategy lies in its relating to gain-loss asymmetry by placing greater utility weight on his or her immediate gains and delayed losses. Although many neuroimaging studies have unveiled the neural mechanisms that underlie moral decision-making, little attention has been paid to the temporal dynamics of how a decision-maker assesses utility weights differently for a moral (or adaptive) choice that will bring loss (or gain) to himself (and others) when the outcome will be realized in the near versus distant future. This study identifies the electrophysiological mechanisms of time-dependent assessment in individuals’ moral conflict resolution strategies. Twenty-two participants were given a set of moral dilemmas with time intervals that varied from the near future to the distant future. Participants chose between two conflicting options: a self-interest-seeking immoral choice (adaptive) and a principled moral choice (moral). Event-related potentials (ERPs) were recorded, and movement-related potentials (MRPs) were analyzed by being response-locked to individual moral choices. Behavioral results showed that participants took more time to respond and were more likely to make adaptive choices under the near-future condition. When the participants faced moral dilemmas, their brain waves manifested medial frontal negativity (MFN) at early stage ERP of 200–400 ms, possibly reflecting an internal moral conflict. Participants then exhibited larger late positive potentials (LPP) under the near-future condition. In addition, greater effort in implementing motor preparation was found under the near-future condition than under the distant future condition, as supported by the larger Bereitschaftspotential (BP) in the anterior areas. Our results illustrate the temporal dynamics of the electrophysiological mechanisms that underlie time-dependent assessments in moral decision-making, as human brains discount the decision utility of the moral outcomes that will occur in the distant future.

## Introduction

Moral dilemmas occur when a decision between two undesirable options must be made (Sinnott-Armstrong, 1987). Researchers in moral psychology have suggested that the degree to which humans’ moral decisions are based on situational factors should be clarified (Bartels et al., 2014), but human moral decisions-making is complicated.

When decisions involve a conflict in moral values, on what do individuals base their decisions? According to deontological moral theory, individuals must do the right thing at all times at all costs (Kant, 1785/1959), but real moral decisions are malleable in nature, as they are influenced by external situational factors (Bartels, 2008). Neuroimaging studies on moral dilemmas integrate the discourse about “neuroeconomic” models of utility to explain how situational contexts account for adaptive decision-making (Platt and Huettel, 2008; Shenhav and Greene, 2010). For example, moral resolution may be prone to framing (Kern and Chugh, 2009; Kahneman and Tversky, 2013), magnitude and probability outcomes (Shenhav and Greene, 2010), and temporal discounting (Böhm and Pfister, 2005).

Intertemporal choice theory in decision-making research refers to how people measure the costs and benefits of a decision at different times before making the final decision (Loewenstein et al., 2003), resulting in differing utility weights for the event around the decision (Thaler, 1985). A fundamental finding of intertemporal choice research is the gain-loss asymmetry phenomenon, where people tend to prefer immediate gains over losses and delayed losses over gains of the identical magnitude (Shelley, 1994). Thus, the phenomenon, also known as the “sign effect”, occurs when the disparity between people’s valuation of gains and losses is asymmetrically enlarged. The sign effect suggests that people asymmetrically discount the utility of future outcomes because they perceive immediate pleasure or pain as more intense than later (Caruso et al., 2008).

Studies have shown that virtually all living species tend to satisfy their current interests before their long-term interests (Chapman, 1996; Löckenhoff et al., 2011), but one of the essential differences between human beings and other animals is that humans can calculate and plan for related events in the future (Humphrey, 1976). Still, when it comes to intertemporal choices that are associated with gain and loss, research has indicated that individuals prefer less immediate gain over more future gain (Ainslie, 1975; Kirby et al., 1999; Wittmann and Paulus, 2008). Therefore, because of the need for exploration of the origin and mechanism of human decisions, time-dependent evaluation of gains and losses has become an important research topic in neuroeconomics. However, studies on individuals’ decision-making as it relates to the adaptive assessment of the decision-maker’s gains and losses are limited. As individuals live in social groups, the choices made in daily life may affect not only themselves but also others (Gardner and Steinberg, 2005; Páez et al., 2008), so research on moral decision-making that involves a tradeoff between the individual’s potential gain or loss and that of others requires additional exploration.

The current study addresses the role of time-dependent assessments in deciding between conflicting outcomes in moral decision-making. This effort is significant in two ways. First, traditional moral dilemmas often involve outcomes that harm others (i.e., killing one person to save five people) (Greene et al., 2001, 2004; Borg et al., 2006), but the outcomes of moral decisions in reality link one’s own gain or loss with another’s gain or loss. For example, imagine a personnel manager who has just found out that her boss, a close friend, could be imprisoned for breaching the code of ethics. The chief prosecutor, another friend, has asked the manager to hire his son, who is not well qualified. Assuming that she has two unwanted options to make, should she accept the prosecutor’s request or not? Doing this favor will result in the manager’s being promoted and will ease the investigation of her boss (i.e., pleasurable outcome), while not doing the favor will not get her promoted and her boss will likely be imprisoned (i.e., painful outcome). Thus, decision outcomes often involve an interpersonally dependent tradeoff. Electrophysiological research on how the brain computes conflicting outcomes in the choice between pursuing one’s self-interest at the cost of others’ is far from well understood. The second way in which our research is significant lies in its relating to gain-loss asymmetry’s view that delayed losses have larger impacts on utility than do delayed gains, while immediate gains have larger impacts on utility than do immediate losses (Shelley, 1994) and asking whether the decision differs if the gains and losses occur in the near future or in the distant future. Neuroscientific studies have not considered the neural correlates of evaluating outcomes in which decision-makers assess both gains and losses based on their distance in time from the near future to the distant future.

Research has linked moral decision-making processes with the lateral frontal cortex and the medial frontal cortex. For example, cognitive reasoning increases activity in the lateral frontal cortex, thus playing a part in suppressing immediate adverse emotional responses (Greene et al., 2004). People with lesions in the ventromedial prefrontal cortex (vmPFC) have shown significantly higher proportions of utilitarian judgments in high-conflict dilemmas (Greene, 2007; Koenigs et al., 2007; Moll and de Oliveira-Souza, 2007; Moretto et al., 2010). Although emotional and utilitarian evaluations of moral dilemmas activate distinctive brain regions, moral value has been found to be represented in the vmPFC (Hutcherson et al., 2015). The medial frontal cortex is also involved both in cognitive conflict (Greene et al., 2004) as well as affective processing (Decety and Cacioppo, 2012), particularly when individuals make difficult moral decisions. Decision conflict that is linked to motor responses when choosing a set of attractive or unattractive options has been observed in the anterior cingulate cortex (ACC) (Pochon et al., 2008). The Stroop and ultimatum game tasks that engage high levels of cognitive conflict have also illustrated the importance of ACC activation (Botvinick et al., 2001; Sanfey et al., 2003). Together, such findings have generated significant research interest in the neural correlates of moral conflict in moral decision-making.

The pivotal roles of the frontal cortex have been highlighted in both moral decision-making (Greene et al., 2001, 2004; Moll and de Oliveira-Souza, 2007; Hutcherson et al., 2015) and time-dependent assessment (McClure et al., 2004; Hare et al., 2014). The frontal cortex particularly have been related to moral conflicts that require cognitive effort (Greene et al., 2001, 2004; Jeurissen et al., 2014; Zheng et al., 2018). The brain regions that are associated with gain-loss asymmetry are the prefrontal cortex and the limbic system, which calculate the relative reward values based on distance in time (McClure et al., 2004; Ballard and Knutson, 2009; Tanaka et al., 2014). For instance, the connectivity from the dorsolateral prefrontal cortex (DLPFC) to the vmPFC has been found to be critical in the decision to delay long-term rewards (Hare et al., 2014). In doing so, we designed a moral task in which participants face moral dilemmas and discount the utility of opposing outcomes (i.e., gains vs. losses) over time.

Although many neuroimaging studies have articulated the neural underpinnings of moral decision-making (Greene et al., 2001, 2004; Borg et al., 2006; Decety and Cacioppo, 2012; Feldmanhall et al., 2014; Hutcherson et al., 2015), there is a dearth of event-related potential (ERP) research on moral decision-making that has incorporated with an economic approach. More specifically, little is known about how individuals asymmetrically discount utility about outcomes in moral judgments based on the distance in time, from the near future to the distant future. ERP is used because it involves the temporal resolution with which to assess the neural correlates of gain-loss asymmetry that underlie decision outcomes in real time (Martin and Potts, 2009; Cherniawsky and Holroyd, 2013; Qu et al., 2013). The ERP study also facilitates the measurement of cortical potentials at two distinct phases of moral decision-making that are time-locked to the choice phase and to the behavioral response (Chen et al., 2009; Sarlo et al., 2012).

We hypothesize that, when the time in which the choice is made is close to the present, participants pay more attention to their immediate gain acquisition and to loss avoidance. Since immediate gains seem more attractive to oneself than immediate losses, they would be more likely to choose adaptive choices (self-interest-seeking). But when the choice is made is distant from the present, they pay more attention to their delayed losses that are less steeply discounted than delayed gains. Thus, they would be more likely to choose moral choices (principled moral) despite having losses. Behaviorally, we hypothesize that the near-future condition evokes a larger proportion of adaptive choices and slower responses times than occur in the distant-future condition. For ERP, we suggest that, when participants make choices, the medial frontal negativity (MFN) at the early ERP components (200–400 ms) are generated at the initial stage of the decision-making progress, possibly predicting moral conflict. The MFN has been considered to be the early component induced by error-related processing (Boksem et al., 2011) and social conflicts (Huang et al., 2014) over the medial frontal sites. However, because of the choice to make the most self-interested choice, motivated engagement in adaptive decisions eventually would occur in the near-future condition, as reflected in late positive potentials (LPP). The later processing component, LPP, generally begins around 300–400 ms after the onset of the stimuli; that is associated with cognitive control (Ruchkin et al., 1982) and motivated attention (Gable and Harmon-Jones, 2010). Therefore, participants would discount the utility of the combined outcomes of gains and losses from the distant future, which would prompt adaptive decision-making in the near-future condition. In addition, the greater Bereitschaftspotential (BP) cortical positivity elicited by the near-future condition would be observed for motor readiness before the participant makes actual behavioral responses (movement-related potentials, MRP). All ERPs and MRPs are reported over the frontal sites as the possible sources in resolving moral dilemmas.

## Materials and Methods

### Participants

Twenty-five right-handed undergraduate and graduate students (9 women) were paid to participate in this experiment. Aged from 19–29 years (mean age 23), participants were native Korean speakers, had normal or corrected-to-normal vision, and had no history of neurological problems. The procedure was approved by the Institutional Review Board (IRB) of Sungkyunkwan University. All participants provided written informed consent prior to participating in the experiment. Three participants’ data were discarded because of an excessive number of recording artifacts, leaving twenty-two valid participants for the final data analysis.

### Stimuli and Procedure

**FIGURE 1:**
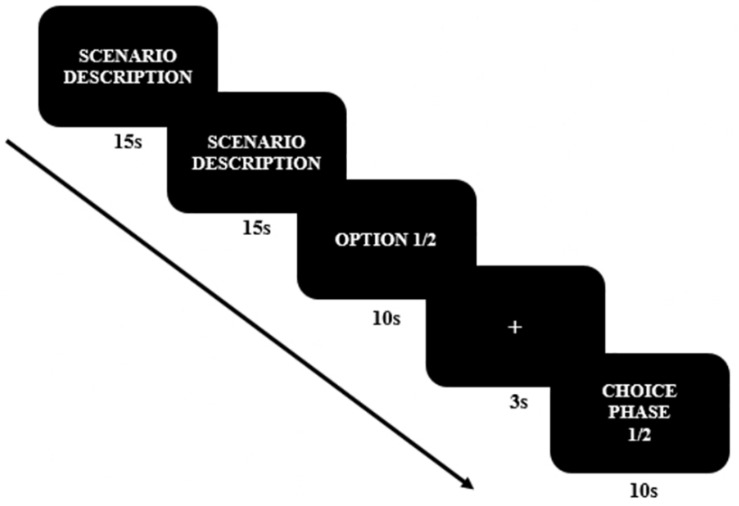
Electroencephalography (EEG) Experiment Paradigm. Both the ERP and the MRP were analyzed at the choice phase.

The electroencephalography (EEG) experiment consisted of a one-factor (temporal distance: the near future vs. the distant future) within-subject design. Each condition was randomly repeated three times, varying the time allowed for decision-making each time (near future: three trials, distant future: three trials), resulting in a total of six trials. We developed one type of moral dilemma (Table 1), and the experimental paradigm was conducted in the sequence shown in Figure 1. The moral dilemmas were presented as white text (25-point Arial) against a black background. The first and second slides described the scenario, and the third slide presented two undesirable options for 10 s. After participants read the descriptions of scenarios and the given options, a baseline brain activity was measured for 3 s. The degree of temporal distance varied from one to 3 days for the near-future condition and from 1–3 years for the distant-future condition. These time frames were stated at the choice phase (e.g., “you have to make this decision in a *day*”). Finally, participants chose between the two balanced options, one an adaptive option (i.e., seeking self-interest) and one a moral option (i.e., principled moral). All six moral dilemmas were randomly presented on a 28-inch computer screen at a viewing distance of 50 cm. The scenario presentation was programmed with E-prime 2.0 software (Psychology Software Tools, Pittsburgh, PA, United States).

**TABLE 1:** Moral dilemma scenario used in the experiment.

**Time**	**Scenarios**
You have to make this decision in:	You are the personnel manager at company A. You have found out that chief prosecutor B, who is a close friend, will be investigating a case of embezzlement against your company’s CEO. Meanwhile, chief prosecutor B’s son is applying for a job in your company Although the son of prosecutor B has lower qualifications than other applicants, the embezzlement investigation is expected to be finished in the boss’s favor if you hire B’s son.
Near future: 1/2/3 Days	– Moral Choice (principled moral): If you decide not to hire Mr. B’s son, the prosecution’s investigation will be tough. You will not be promoted to managing director, and your company”s CEO will be imprisoned.
Distant future: 1/2/3 Years	– Adaptive Choice (self-interest-seeking immoral): If you decide to hire Mr. B’s son, the prosecution’s investigation will go in your CEO’s favor, and you will be promoted to managing director.

The EEG experiment for each participant was scheduled in advance. Each participant was brought individually into a laboratory, a sound-attenuated, dimly lit room, and instructed on how the experiments would be conducted. Instructions on handouts were provided and additional explanation was delivered orally. Participants were seated in a comfortable chair while the experimenter attached the EEG electrodes to the participant’s scalp.

Each dilemma started with the three-slide description of the scenario, which participants read on the computer screen. Participants were instructed to indicate their final decision by pressing a button (1 or 2) on a response pad. (They were told to wait for the choice slide before indicating their final decision.) To minimize finger-movement artifacts, participants were asked to keep the index and middle fingers of their right hands above the response pad, to maintain their body position, and to refrain from blinking as much as possible throughout the task. After each response, an intertrial interval of 3 s (i.e., fixation) passed before the next dilemma began. The intertrial periods acted as a control (filler) condition. The whole EEG experiment tasks took about 20 min, and then participants completed the post-survey for the manipulation check (Q: how imminent did the need to make a decision feel? 1 = Not at All; 7 = Very). Participants were paid $30, thanked, and debriefed.

### EEG Recordings and Analysis

Electroencephalography data were recorded using a 64-channel MR-compatible EEG system (Brain Products GmbH, Germany). Sixty-three Ag/AgCL electrodes (FP1, FP2, AFz, AF3, AF4, AF7, AF8, Fz, F1, F2, F3, F4, F5, F6, F7, F8, FCz, FC1, FC2, FC3, FC4, FC5, FC6, FT7, FT8, FT9, FT10, Cz, C1, C2, C3, C4, C5, C6, T7, T8, CPz, CP1, CP2, CP3, CP4, CP5, CP6, TP7, TP8, TP9, TP10, Pz, P1, P2, P3, P4, P5, P6, P7, P8, POz, PO3, PO4, PO7, PO8, Oz, O1, and O2) were placed on an elastic cap (actiCap, Brain Products GmbH) according to the standard international 10/20 system. A syringe was used to inject the electroencephalogram gel (EEG Gel Supervisc.) into all sixty-three channels embedded in the cap. The FCz channel located at midline frontal-central was selected as the reference channel, and the AFz channel located at the frontopolar was selected as the ground channel. All electrode impedances were maintained below 10 Ω during the recording. The EEG signals were continuously sampled at the 500 Hz/channel rate and were amplified by a Brain Products GmbH MR amplifier (BrainVision antiChamp, Gilching, Germany) using a 0.01 to 100 Hz band-pass filter.

Further data analyses processing was done using EEGLAB and ERPLAB (Delorme and Makeig, 2004; Lopez-Calderon and Luck, 2014) on Matlab (Mathworks, NJ). The BSS-based electro-oculograms (EOG) procedure was applied to correct ocular artifacts with a fully automated approach produced by SOBI (Second-Order Blind Identification) (Gómez-Herrero et al., 2006). The method enables the researcher to detect ocular movements and movement-related artifacts without necessarily attaching EOG reference channels. Both time-locked to the choice phase (ERP) and to the behavioral response (MRP) were processed and analyzed at the choice phase. To compute ERPs, continuous EEG was segmented off-line into 900-msec epochs from 100 msec before to 1000 msec after the onset of the choice phase. To compute MRPs, continuous EEG was segmented off-line into 1500-msec epochs from 1000 msec before to 500 msec after the behavioral response (1/2 keypress). ERPs and MRPs were filtered between 0.1 and 20 Hz low-pass (24 dB octave roll off). All EEG epochs were then baseline-corrected during the 100-msec prestimulus period for ERP and during the 200-msec period from −1000 to −800 msec preceding the keypress for MRP. All epochs were visually scored for artifacts, and EEG voltage amplitudes that exceeded a threshold of ±75 μV during the recording were rejected and excluded from the final analysis. Thus, artifact-free trials were separately averaged for each participant in each condition, resulting in a total of 88.5 percent and 89.7 percent for the near- and distant-future conditions, respectively. Furthermore, the control condition (i.e., an intertrial interval), which was averaged across all six trials, was analyzed and reported only during the ERPs since the intertrial interval was presented after the response onset.

Based on visual inspection of grand-averaged ERP waveforms and temporal distance conditions, one prominent negativity was observed between 200 and 400 msec (early negativity) after the onset of the choice phase. In addition, condition effects of late potentials were clearly discernible in the time interval after 400 msec, so the mean amplitude for the time windows of 400–600 msec and 600–800 msec (LPP) were analyzed separately.

Based on the EEG study (Sarlo et al., 2012), visual inspections of grand-averaged MRP waveforms were reflected in the analysis of the two separate time windows: the mean amplitudes of MRP components between 800 and 600 msec before keypress, referred to as early BP, and those between 500 and 100 msec before keypress, referred to as late BP.

The proportion of adaptive choices was calculated by dividing the number of adaptive choices by the total number of adaptive and moral choices for each condition. Two-tailored *t*-tests were run to compare the response times and the proportion of adaptive choices between the two manipulated conditions.

Two-way repeated-measure analyses of variances (ANOVAs) were employed to determine the mean amplitudes for early negativity in ERP and for late positivity in ERP. The mean amplitudes for both early and late BPs were analyzed in MRP. During the time window of interest, fourteen electrode sites over the frontal regions (AFz, AF3, AF4, AF7, AF8, Fz, F1, F2, F3, F4, F5, F6, F7, and F8) were selected for all the analyses in ERPs and MRPs. The Greenhouse-Geisser correction was applied to all *F* ratios for effects within factors with more than two levels when the sphericity assumption was violated. For the *post hoc* evaluations, a Bonferroni-corrected *p*-value was used to identify significant main effects.

## Results

### Behavioral Data

For the manipulation check, all twenty-two participants reported that the near-future condition felt more imminent before they made a final decision than the distant-future condition did (*t*(21) = 5.07, *M*Near = 4.87, *M*Distant = 1.87, *p* = 0.018).

The near-future condition led to a significantly higher proportion of adaptive choices than the distant-future condition did (*t*(21) = 4.11, *M*Near = 42%, *M*Distant = 32%, *p* < 0.001) and elicited longer response times (*t*(21) = 8.01, *M*Near = 2546 ms, *M*Distant = 1939 ms, *p* < 0.0001).

### ERP Results

**FIGURE 2:**
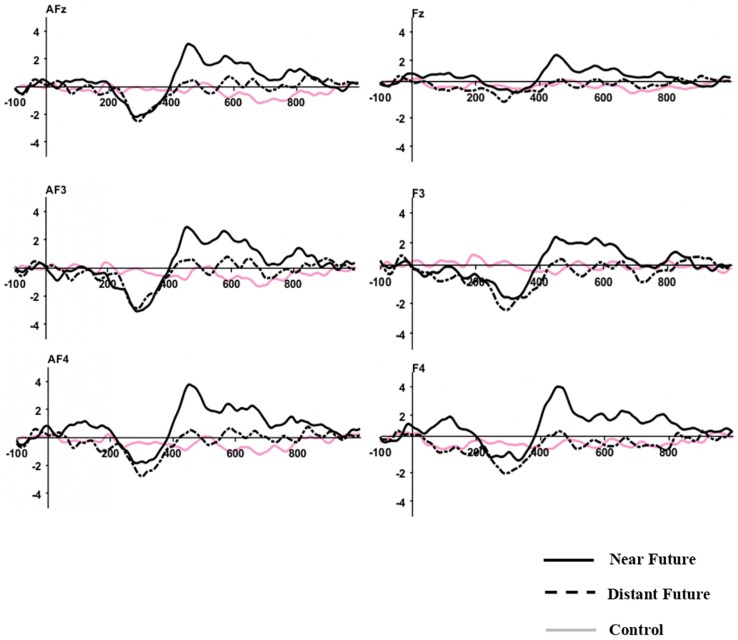
Grand-Averaged ERP Waveforms. Time 0 indicates the onset of the choice phase. The Y-axis indicates amplitude (μV).

Grand-averaged ERP waveforms elicited by both temporal conditions after the onset of the choice phase are depicted at frontal-midline sites in Figure 2.

#### Early Negativity (200–400 msec)

After applying the Greenhouse-Geisser correction, the repeated-measure ANOVA for mean amplitudes at frontal locations during 200–400 ms revealed no significant main effect of temporal distances (*p* = 0.181). There were also no significant differences for the main effect of electrodes and interaction between two effects (*p* > 0.05). *Post hoc* comparisons affirmed that the mean amplitudes for the distant-future condition (*M* = −1.69 μV) were slightly higher than those of the near-future condition (*M* = −1.09 μV) and the control condition (*M* = −0.21 μV). Such differences were also not significant at other locations (i.e., fronto-central, central, centro-parietal, temporal, parietal, and occipital sites).

#### Late Positivity (400–600 and 600–800 msec)

After applying the Greenhouse-Geisser correction, the repeated-measure ANOVA for mean amplitudes at frontal locations during 400–600 ms revealed a significant main effect of temporal distance [*F*(2,42) = 5.334, *p* = 0.012, η2 = 0.203]. No significant differences were found for the main effect of electrodes and interaction between two effects (*p* > 0.05). *Post hoc* comparisons confirmed that the mean amplitudes for the near-future condition (*M* = 2.03 μV) were more positive than those of the distant-future condition (*M* = 0.12 μV) and the control condition (*M* = −0.38 μV). However, this difference was not significant at other locations (i.e., fronto-central, central, centro-parietal, temporal, parietal, and occipital sites).

In the time window of 600–800 ms revealed a marginal significant main effect of temporal distance [*F*(2,42) = 2.616, *p* = 0.087, η2 = 0.111]. No significant differences were found for the main effect of electrodes and interaction between two effects (*p* > 0.05). *Post hoc* comparisons confirmed that the mean amplitudes for the near-future condition (*M* = 1.30 μV) were more positive than those of the distant-future condition (*M* = 0.02 μV) and the control condition (*M* = −0.42 μV). However, this difference was not significant at other locations (i.e., fronto-central, central, centro-parietal, temporal, parietal, and occipital sites).

### MRP Results

**FIGURE 3:**
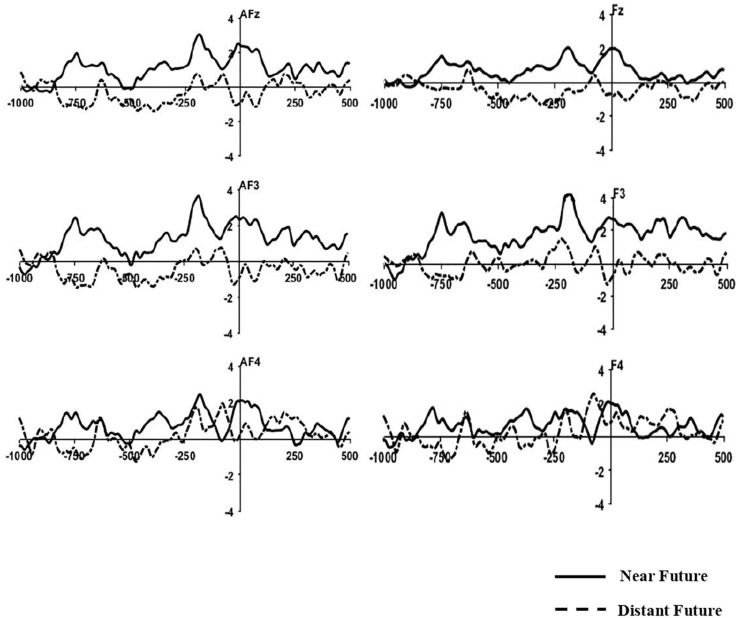
Grand-Averaged MRP Waveforms. Time 0 indicates the onset of the behavioral response. The Y-axis indicates amplitude (μV).

Grand-averaged ERP waveforms elicited by both temporal conditions before response onset are depicted at frontal-midline sites in Figure 3.

#### Early BP (From 800 to 600 msec Before Response Onset)

After applying the Greenhouse-Geisser correction, the repeated-measure ANOVA for mean amplitudes at frontal locations during 800–600 ms revealed a significant main effect of temporal distance [*F*(2,42) = 7.730, *p* = 0.011, η2 = 0.269]. No significant differences were found for the main effect of electrodes and interaction between two effects (*p* > 0.05). *Post hoc* comparisons confirmed that the mean amplitudes for the near-future condition (*M* = 1.16 μV) were more positive than those of the distant-future condition (*M* = −0.53 μV). However, this difference was not significant at other locations (i.e., fronto-central, central, centro-parietal, temporal, parietal, and occipital sites).

#### Late BP (From 500 to 100 msec Before Response Onset)

After applying the Greenhouse-Geisser correction, the repeated-measure ANOVA for mean amplitudes at frontal locations during 500–100 ms revealed no significant main effects of temporal distance (*p* = 0.076). No significant differences were also found for the main effect of electrodes and interaction between two effects (*p* > 0.05). Yet, *post hoc* comparisons showed greater positivity for the near-future condition (*M* = 1.11 μV) than the distant-future condition (*M* = −0.09 μV). This difference was also not significant at other locations (i.e., fronto-central, central, centro-parietal, temporal, parietal, and occipital sites).

**FIGURE 4:**
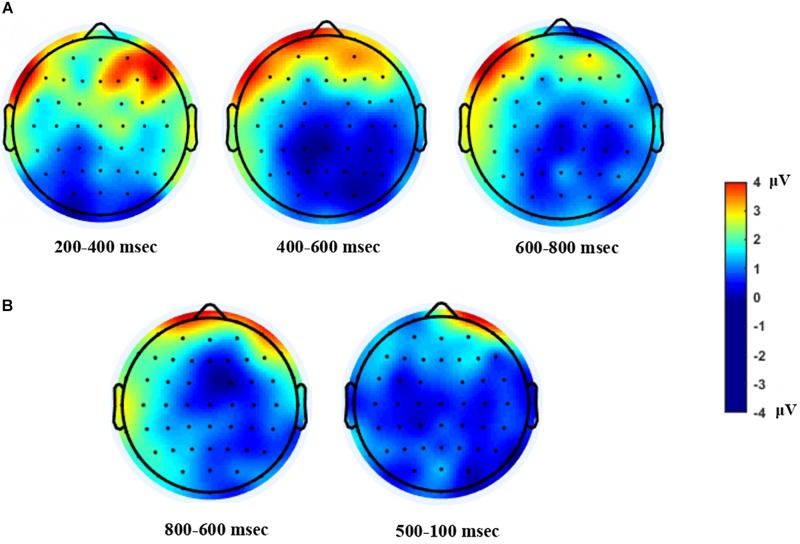
Topographical Maps. Topo scalp distribution of the difference waves, computed by subtracting the distant-future condition from the near-future condition. **(A)** ERP topo is shown after the onset of the choice phase. **(B)** MRP topo is shown before response onset. The greater anterior positivity displayed in both the ERP and the MRP analyses indicates higher positivity for the near-future condition relative to the distant-future condition.

To summarize the ERP and MRP results, mean amplitude differences between the near-future and distant-future conditions in each specific time window are shown by topographical maps in Figure 4.

## Discussion

Our results show how human brains discount utility in considering conflicting outcomes that occur from the near future to the distant future. Drawing on economic models, the current research examines the electrophysiological correlates of time-dependent assessments that are responsible for adaptive decision-making in moral dilemmas. Our findings of moral decision-making address the long debate between deontological and utilitarian theories. We expand the purview of moral decision-making, as judgments are not only understood using moral principles but are also characterized by flexible systems so they can adapt to a situation in the near future (Bartels et al., 2014). Our results are notable for two reasons: First, unlike traditional moral dilemmas that concern identical life-and death outcomes for others, we explain how moral conflicts can occur for the decision-maker in evaluating outcomes that are characterized by gains (i.e., self-interest-seeking) as opposed to losses (i.e., moral principles). Second, participants are found to devalue non-monetary utility related to pleasant and painful outcomes that occur in the distant future, which leads them to adopt adaptive decision-making in the near future. Previous neuroimaging studies have used monetary rewards to see how individuals discount their relative value at diverse points in time (McClure et al., 2004; Hare et al., 2014; Tanaka et al., 2014). Another ERP study used real money and found larger feedback-related negativity (FRN) between loss and gain with immediate outcomes (Qu et al., 2013). We also note that using hypothetical decision outcomes (i.e., being promoted or imprisoning the boss) that occur from the near future to the distant future produces distinct neural correlates of gain-loss asymmetry. Participants tend to avoid painful losses and to choose pleasant gains in the near future more often than they do in the distant future (Caruso et al., 2008). In light of these findings, we explored the electrophysiological correlates of gain-loss asymmetry at separate time phases of moral decision-making (i.e., ERP and MRP).

The behavioral results are consistent with the extant research on moral dilemmas in three ways. First, we found that the proportion of adaptive choices was significantly higher in the near future than it was in the distant future, while Sarlo et al. (2012) ERP study showed that participants made a higher proportion of adaptive choices in responding to decisions involving unintended consequences. Second, we found that the response times were significantly slower in the near-future scenarios than they were in the distant-future scenarios, while Greene et al. (2001) fMRI study reported slower reaction times for adaptive decisions in moral-personal dilemmas that accompanied emotional engagement. Third, temporal distance has been argued to influence moral decision-making, leading to moral incompetency (Agerström and Björklund, 2009a, b; Suter and Hertwig, 2011; Lee and Yun, 2019); we found that participants made decisions slowly and favored self-interested decisions in the near-future condition.

Time-locked to the onset of the choice phase in ERP, ostensible potential was found for both temporal conditions, peaking the MFN. On the basis of the topographical maps, we might speculate that such observations were prominent over the frontal sites. When participants processed monetary gains and losses, only losses evoked the early negativity generated by a medial-frontal activity (Gehring and Willoughby, 2002). MFN was also apparent when recipients were subjected to unfair bargaining in gambling situations (Boksem and De Cremer, 2010). Similarly, brain activations in MFN have been associated with social conflicts (Huang et al., 2014) and processing “error” signals from the social environment, particularly among low-status individuals (Boksem et al., 2011). Moreover, the FRN in the early stage of outcome assessment, sourced in the medial prefrontal areas, has been regarded as an effective signal of neural correlates of delay discounting (Qu et al., 2013). Our results report similar patterns, as both time conditions elicited the MFN found in ERP analyses. Since moral resolutions of dilemmas naturally demand strenuous effort, irrespective of temporal conditions, we considered MFN a trigger of moral conflict before evaluating the decision outcome.

Another component identified after the onset of the choice phase in ERP is LPP for the near-future condition. Following MFN for both conditions, we analyzed two time windows, one at 400–600 ms and one at 600–800 ms. The near-future condition elicited greater cortical positivity at more frontal locations than the distant-future condition did. In particular, the bilateral activity of the lateral frontal cortex is known to involve rational cognitive control in resolving moral-personal dilemmas (Greene et al., 2001, 2004). Furthermore, LPP has been associated with the cognitive resources required during later processing (Ruchkin et al., 1982), motivational significance to affection (Schupp et al., 2000; Lang and Bradley, 2010), and allocation of attention to luxury brands (Pozharliev et al., 2015). Similarly, utilitarian decisions on incidental dilemmas that described an expected but unintended outcome showed LPP at posterior sites that required cognitive resources (Sarlo et al., 2012). However, our results found the LPP at anterior sites, particularly lateralized to the left hemisphere (Figure 4). Using the three-way repeated-measure ANOVA, we conducted an additional analysis to see the effect of frontal asymmetry on the mean amplitudes of four electrode sites (AF7, AF8, F7, and F8), temporal distance (the near future and the distant future), and laterality (left and right) as within-subject factors during the 600–800 ms time period. Although we found no statistical differences between the left (AF7 and F7) and the right electrode locations (AF8 and F8) during the 200–400 ms and 400–600 ms time periods (*p* > 0.05), we found a significant effect of temporal distance [*F*(2,42) = 7.498, *p* < 0.05, η2 = 0.26] and laterality [*F*(2,42) = 4.352, *p* = 0.049, η2 = 0.17] during the 600–800 ms time period. In laterality left, the LPP amplitude for the near-future condition (*M* = 2.77 μV) was significantly higher than that for the distant-future condition (*M* = −1.14 μV), while there were no significant differences between temporal distances in laterality right (*p* > 0.05). We investigated asymmetrical motivational processing (Gray, 2001; Coan and Allen, 2004), as previous work found that the greater left frontal LPP amplitudes to appetitive stimuli are related to motivated attention (Gable and Harmon-Jones, 2010). We found increased approach motivational engagement in the near-future condition and reduced approach motivational engagement in the distant-future condition. Taking these findings together, we conclude that the near-future condition yields left frontal LPP, as participants felt motivated to decide after resolving a moral conflict by including additional attentional resources.

We analyzed response-locked MRP in two consecutive time windows to articulate the second phase of decision-making. The early BP and late BP components were apparent for the near-future condition over the frontal areas. The BP involves the preparatory processes that precede the implementation of self-initiated movement, which is reflected in an increase in cortical excitability (Shibasaki and Hallett, 2006). The early BP at the time window of 800–600 ms was observed before the movement onset in the pre-supplementary motor area (pre-SMA) (Cunnington et al., 1996). The late BP at the time window of 500–100 ms occurred just before the behavioral response, representing the activity of the contralateral premotor and primary motor cortex (Ikeda et al., 1992). The late component specifies features of movement execution. These results are in line with the results of previous neuroimaging investigations that have found more SMA activation under the moral dilemmas that involve unintentional harm (Borg et al., 2006; Sarlo et al., 2012), and with the results of another ERP study that also found N2 during counter-conformity purchase decisions in the ACC and pre-SMA (Gajewski et al., 2016). Larger BP cortical potential elicited by the near-future condition accompanied greater effort in preparing to execute the actual behavioral response.

## Conclusion

In conclusion, our findings provide insights into the link between gain-loss asymmetry and moral decision-making. A resolution strategy when a person is faced with moral dilemmas may resort to gain-loss asymmetry, as a decision-maker tends to put greater weight on immediate gains and delayed losses than on the reverse. The ERP results show that moral resolutions of dilemmas evoked the MFN, regardless of the temporal distance condition. However, the near-future condition elicited LPP, as participants were motivated to engage in adaptive decisions after resolving moral conflict. The LPP was observed in the anterior sites, as the near-future condition required additional cognitive resources and effort. The MRP results also suggest that larger BP amplitude detected under the near-future condition indicate greater pre-SMA and motor readiness before the participants made final decisions. Therefore, moral judgment recruits time-dependent mechanisms, as humans devalue utility in assessing conflicting outcomes in the distant future.

The study has several limitations that lead to suggestions for future research. First, virtual moral situations using vignettes contain issues related to external validity. Although such issues are commonly found in experimental settings, future research could use a more realistic setting. Second, reading imagined vignettes while the EEG recordings were made could be difficult for participants, as they were instructed to maintain body motion and refrain from blinking too much. We acknowledge that constraints to scenario-based experimental design using the ERP should be addressed. Since reading stimuli involves dynamic processes, we placed the decision slide in the last position so participants could finish reading it before making their final choices. Third, insufficient robustness of the current findings, given the number of trials presented per condition (i.e., three trials), might have limited statistical power. However, we had to be cautious about any possible suppression or demand effect since we used an identical dilemma scenario across all six trials. Future research could use more than five trials per condition that will increase confidence in generalizing our results. Finally, although the temporal part of the brain is a core region in moral tasks (Jeurissen et al., 2014; Haas et al., 2015; Gan et al., 2016), we did not find a main effect of temporal distance in the temporal-related areas. Our dilemma task did not involve life-and-death outcomes that harm others but examined the mechanism that underlies time-dependent assessments in considering combined outcomes of gains and losses at different times. In addition, altering the excitability of the bilateral TPJ regions has been found to have little impact on moral judgments (Zheng et al., 2018). Despite these limitations, our findings help to clarify yet another feature of moral decision-making by integrating the economic model of gain-loss asymmetry into our research.

## Data Availability Statement

The raw data supporting the conclusions of this manuscript will be made available by the authors, without undue reservation, to any qualified researcher.

## Ethics Statement

The whole procedure was approved by the Institutional Review Board (IRB) of Sungkyunkwan University, South Korea.

## Author Contributions

JY and E-JL designed the experiments. JY and JZ conducted the experiments and collected the behavioral and ERP data. JY processed and analyzed the data. JY wrote the main manuscript under the supervision of E-JL and all authors reviewed it.

## Conflict of Interest

The authors declare that the research was conducted in the absence of any commercial or financial relationships that could be construed as a potential conflict of interest.
